# Cystic Fibrosis Kidney Disease: 10 Tips for Clinicians

**DOI:** 10.3389/fmed.2018.00242

**Published:** 2018-08-28

**Authors:** Alexandra C. H. Nowakowski

**Affiliations:** Geriatrics, Behavioral Sciences and Social Medicine, Florida State University College of Medicine, Tallahassee, FL, United States

**Keywords:** cystic fibrosis, aging, illness management, patient perspectives, best practices

## Abstract

Increased longevity in people with cystic fibrosis (CF) means that more people are surviving long enough to develop kidney complications. Nephrologists and their colleagues now face a steep learning curve as many of them encounter patients with CF related kidney disease (CFKD) for the first time. This article presents perspectives from a medical sociologist with CF on what renal health professionals should know about people with CFKD. It outlines challenges that people with CFKD as they age, framing these struggles as opportunities for clinicians to help these unique patients achieve and maintain their best possible quality of life.

Caring for people who are aging with cystic fibrosis (CF) presents unique challenges for kidney health professionals (1). Many people thriving with CF overall still develop chronic kidney disease as we grow older (2). Managing these issues now constitutes a critical component of healthy aging with CF.

I recently turned 34, which once would have been quite old for a CF patient (3). I am generally doing quite well, but have had symptoms of CF related kidney disease (CFKD) since at least my early 20s. These may include generalized chronic kidney disease (abnormal kidney function) and/or individual renal issues such as: mucus casts in the urine, stones in the tubules, pain in the flanks, fluid retention in the lower body, bacterial infections in the kidneys, nephritis from medication sensitivities, and wasting of electrolytes like potassium (4). Some of these issues may be specific to particular CF management strategies, such as antibiotics—and may not occur in all people treated with those approaches (5).

My symptoms get worse if I drink too much liquid or my electrolyte levels drop. This creates a horrible vicious cycle, because low electrolyte levels often make people very thirsty. Increased thirst leads to higher fluid consumption and thus more electrolyte wasting (6).

I should mention that lack of medical knowledge has never been a barrier for me. I grew up in my parents' neuroscience lab at a medical school, earned multiple advanced degrees in health disciplines, and eventually became a medical educator myself. Despite these advantages, I have found managing CFKD frustrating.

As I have worked with my clinicians to recover from inadequate management of my CF during childhood and adolescence, I have frequently heard similar statements from them. My current nephrologist noted that providers are now learning about management of CFKD largely from patients. This is a newer area of clinical practice because not long ago, people with CF rarely survived long enough to develop kidney disease (7). By consequence, things that kidney health practitioners who treat people with CF observe in their daily practice have not necessarily emerged yet in the research literature on CFKD. This piece thus offers implicit guidance for further study as well as explicit recommendations for direct clinical practice.

As a medical educator who has lived with CFKD for many years, I see value in sharing lessons I have learned. This article primarily reflects my own perspectives as a patient while also incorporating my background as an interdisciplinary sociomedical scientist. It uses input from a small group of clinicians and evidence from the research literature as context for my own voice as a person aging with CFKD. The article thus includes elements of both case study and critical review, but should be viewed principally as a perspectives piece that centers opportunities in both research and practice. Here are my top 10 tips for kidney providers working with adult CF patients:

1. **Talk about my sex life**. Ask me how I work to protect against urinary tract infections (8). Ask detailed questions and make suggestions. Affirm me in pursuing safe sexual activities. Remind me that it is okay to feel uncomfortable doing things that cause pain or infections. I know this now, but you will see many other patients who do not yet.
2. **Take my reports of edema seriously**. Thin people get swelling too. Check my eyeballs for bulging or glassiness. Check the skin over my shinbones to see if it shines. Monitor what my legs look like at different times of day when I come in for appointments. Palpate my stomach, hips, and flanks. Pay attention to changes in my weight—especially on a smaller person, fluid retention can make a big difference (9).
3. **Discuss transplantation with me**. Even if you doubt I will need a transplant any time soon. Do not avoid discussing transplantation just because you know talking about it scares me. Information about long-term outcomes from CF kidney disease remains difficult for patients to access, even those of us who work in the medical field ourselves. The best sources of information available are often discussions with other CF patients who experience kidney complications, and discussions with clinicians who know our own health history and its contexts. Talking honestly about options for the future reminds me that I can still have a good life if my kidneys eventually fail, and helps me envision the potential benefits and drawbacks of transplantation for myself as an individual (10).
4. **Ask about my nutrition**. Even if you discussed it at my last appointment. Managing the nutritional demands of CF is challenging (11). For patients with CFKD, it even more difficult. Check in about how much protein and carbohydrate I consume. Make sure that I am getting good nutrition even on days when I feel very ill.
5. **Assess my electrolyte supplement dosage**. Do this for any supplements I am prescribed. Sometimes a dose that once sufficed to keep me stable proves insufficient as my CFKD progresses (12). Ask about tolerance of supplements and any trouble swallowing them. Help me find the least problematic options for electrolyte maintenance.
6. **Provide me with specimen cups**. This allows me to catch my urine any time I suspect an infection (13). Keep me supplied with lab orders so I can get samples cultured promptly. Make sure I know the time window within which samples must be processed after collection. Having urine declined at the lab is frustrating.
7. **Check in about my blood glucose levels**. The fact that I do not currently have CF related diabetes does not mean that I never will. Some CF patients without clinical diabetes have other issues with blood sugar management (14). Monitor me for any potential kidney impacts from blood sugar issues, not just diabetes.
8. **Find out how much water I am drinking**. People with CFKD have to drink enough liquid to physically thin our mucus and flush out hardened casts from the tubules (4). However, we must also exercise caution not to drink so much that we stress our kidneys (15) or exacerbate any existing electrolyte imbalances (12). We do not always succeed, especially because we frequently feel very thirsty. Make sure I monitor the color of my urine and adjust my fluid intake accordingly.
9. **Inquire about my comfort and pain levels**. A lot of people do not know that people with CF frequently experience pain. Sometimes we get so accustomed to living with pain that we do not attend to it until someone asks us outright (16). If I am not in pain, ask me if I feel fatigued, or if my limbs feel heavy. Watch carefully to see if I wince when you examine me.
10. **Coordinate all care activities with my CF specialist**. Every time. No exceptions. This is crucial for both my physical and mental well-being as well as my work (17). Like many adults with CF, I work full-time and focus on giving back to the chronic illness community. This means I cannot spend all day trying to locate medical records.

Finally, a general suggestion for both seasoned nephrology providers and those who are just starting out. Reading literature and talking to colleagues are always great ways to learn about best practices in kidney care for people aging with CF. However, there will be times when the right strategy still seems unclear. At those times, the absolute best thing to do is ask the patient! Adults with CF are usually accustomed to advocating for ourselves and quite knowledgeable about our bodies (18). Take advantage of that whenever you can.

## Author contributions

The author confirms being the sole contributor of this work and approved it for publication.

### Conflict of interest statement

The author declares that the research was conducted in the absence of any commercial or financial relationships that could be construed as a potential conflict of interest.

## References

[1] Farrell PM White TB Ren CL Hempstead SE Accurso F Derichs N Diagnosis of cystic fibrosis: consensus guidelines from the Cystic Fibrosis Foundation. J Pediatr. (2017) 181:S4–15. 10.1016/j.jpeds.2016.09.064 28129811

[2] Kerem E Cohen-Cymberknoh M. Disparities in Cystic fibrosis care and outcome: socioeconomic status and beyond. Chest J. (2016) 149:298–300. 10.1016/j.chest.2015.08.021 26867826

[3] Alicandro G Frova L Di Fraia G Colombo C. Cystic fibrosis mortality trend in Italy from 1970 to 2011. J Cystic Fibros. (2015) 14:267–74. 10.1016/j.jcf.2014.07.010 25151032

[4] Abramowsky CR Swinehart GL. The nephropathy of cystic fibrosis: a human model of chronic nephrotoxicity. Hum pathol. (1982) 13:934–9. 10.1016/S0046-8177(82)80056-7 7129409

[5] Ewence AE Malone S Nutbourne A Higton A Orchard C 302 A retrospective review of renal function and intravenous (IV) antibiotic use in an adult UK cystic fibrosis centre. J Cystic Fibros. (2017) 16:S139 10.1016/S1569-1993(17)30641-0

[6] Li L Somerset S. Dietary intake and nutritional status of micronutrients in adults with cystic fibrosis in relation to current recommendations. Clin Nutr. (2016) 35:775–82. 10.1016/j.clnu.2015.06.004 26159903

[7] MacKenzie T Gifford AH Sabadosa KA Quinton HB Knapp EA Goss CH. Longevity of patients with cystic fibrosis in 2000 to 2010 and beyond: survival analysis of the cystic fibrosis foundation patient registry: lifetime of patients with cystic fibrosis in 2000 to 2010 and beyond. Ann Intern Med. (2014) 161:233–41. 10.7326/M13-0636 25133359 PMC4687404

[8] Driscoll JA Brody SL Kollef MH. The epidemiology, pathogenesis and treatment of *Pseudomonas aeruginosa* infections. Drugs (2007) 67:351–68. 10.2165/00003495-200767030-00003 17335295

[9] Robertson MB Choe KA Joseph PM. Review of the abdominal manifestations of cystic fibrosis in the adult patient. Radiographics (2006) 26:679–90. 10.1148/rg.263055101 16702447

[10] Siminoff LA Traino HM Genderson MW. Communicating effectively about organ donation: a randomized trial of a behavioral communication intervention to improve discussions about donation. Transplant Direct (2015) 1:e5. 10.1097/TXD.0000000000000513 26146659 PMC4486302

[11] Stallings VA Stark LJ Robinson KA Feranchak AP Quinton H Clinical Practice Guidelines on Growth and Nutrition Subcommittee; *Ad Hoc* Working Group. Evidence-based practice recommendations for nutrition-related management of children and adults with cystic fibrosis and pancreatic insufficiency: results of a systematic review. J Am Diet Assoc. (2008) 108:832–9. 10.1016/j.jada.2008.02.020 18442507

[12] Remuzzi G Perico N Macia M Ruggenenti P The role of renin-angiotensin-aldosterone system in the progression of chronic kidney disease. Kidney Int. (2005) 68:S57–65. 10.1111/j.1523-1755.2005.09911.x 16336578

[13] Moskowitz SM Foster JM Emerson J Burns JL. Clinically feasible biofilm susceptibility assay for isolates of *Pseudomonas aeruginosa* from patients with cystic fibrosis. J Clin Microbiol. (2004) 42:1915–22. 10.1128/JCM.42.5.1915-1922.2004 15131149 PMC404629

[14] Moran A Dunitz J Nathan B Saeed A Holme B Thomas W. Cystic fibrosis–related diabetes: current trends in prevalence, incidence, and mortality. Diabetes Care (2009) 32:1626–31. 10.2337/dc09-0586 19542209 PMC2732133

[15] Bar-Or O Hay JA Ward DS Blimkie CJ MacDougall JD Wilson WM. Voluntary dehydration and heat intolerance in cystic fibrosis. Lancet (1992) 339:696–9. 10.1016/0140-6736(92)90597-V 1347582

[16] Ravilly S Robinson W Suresh S Wohl ME Berde CB. Chronic pain in cystic fibrosis. Pediatrics (1996) 98:741–7. 8885955

[17] Tuchman LK Schwartz LA Sawicki GS Britto MT. Cystic fibrosis and transition to adult medical care. Pediatrics (2010) 125:566–73. 10.1542/peds.2009-2791 20176665

[18] Nowakowski ACH. Hope is a four-letter word: riding the emotional rollercoaster of illness management. Sociol Health Illn. (2016) 38:899–915. 10.1111/1467-9566.12403 26865093

